# Supplementary material for: Body composition predicts poor outcomes and reveals immunometabolic dysfunction via single‐cell profiling in anti‐BCMA CAR T‐treated myeloma
Source: Hemasphere. 2026 Mar 24;10(3):e70314. doi: 10.1002/hem3.70314 (PMC13045474; doi:10.1002/hem3.70314)
Supplement: Supplementary file 1 — Supplement. [file HEM3-10-e70314-s001.pdf]

Fig. S1

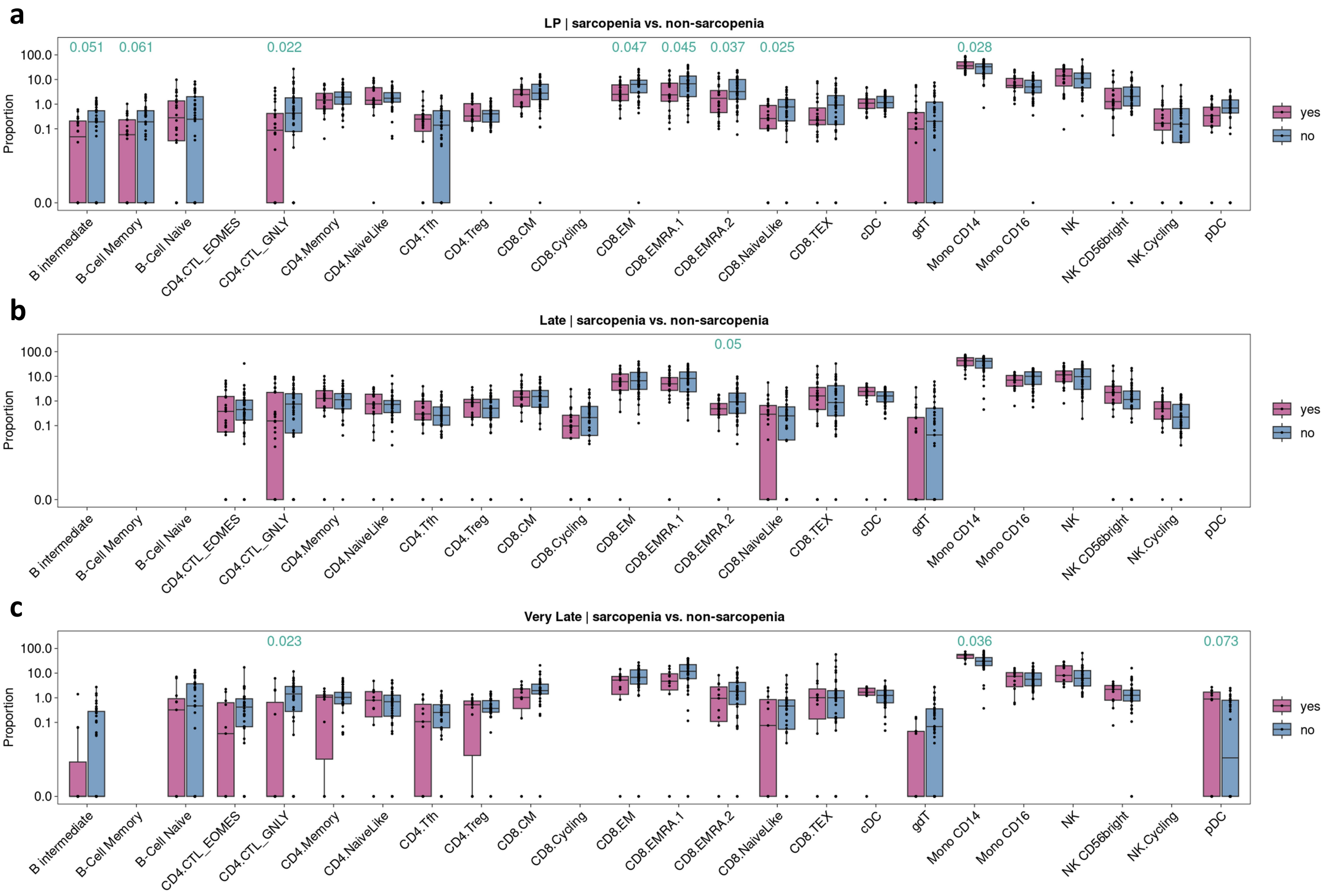
a
LP | sarcopenia vs. non-sarcopenia
0.051 0.061 0.022 0.047 0.045 0.037 0.025 0.028
b
Late | sarcopenia vs. non-sarcopenia
0.05
c
Very Late | sarcopenia vs. non-sarcopenia
0.023 0.036 0.073
Proportion
100.0
10.0
1.0
0.1
0.0
yes
no
B intermediate
B-Cell Memory
B-Cell Naive
CD4.CTL_EOMES
CD4.CTL_GNLY
CD4.Memory
CD4.NaiveLike
CD4.Tfh
CD4.Treg
CD8.CM
CD8.Cycling
CD8.EM
CD8.EMRA.1
CD8.EMRA.2
CD8.NaiveLike
CD8.TEX
cDC
gdT
Mono CD14
Mono CD16
NK
NK CD56bright
NK.Cycling
pDC

Fig. S2

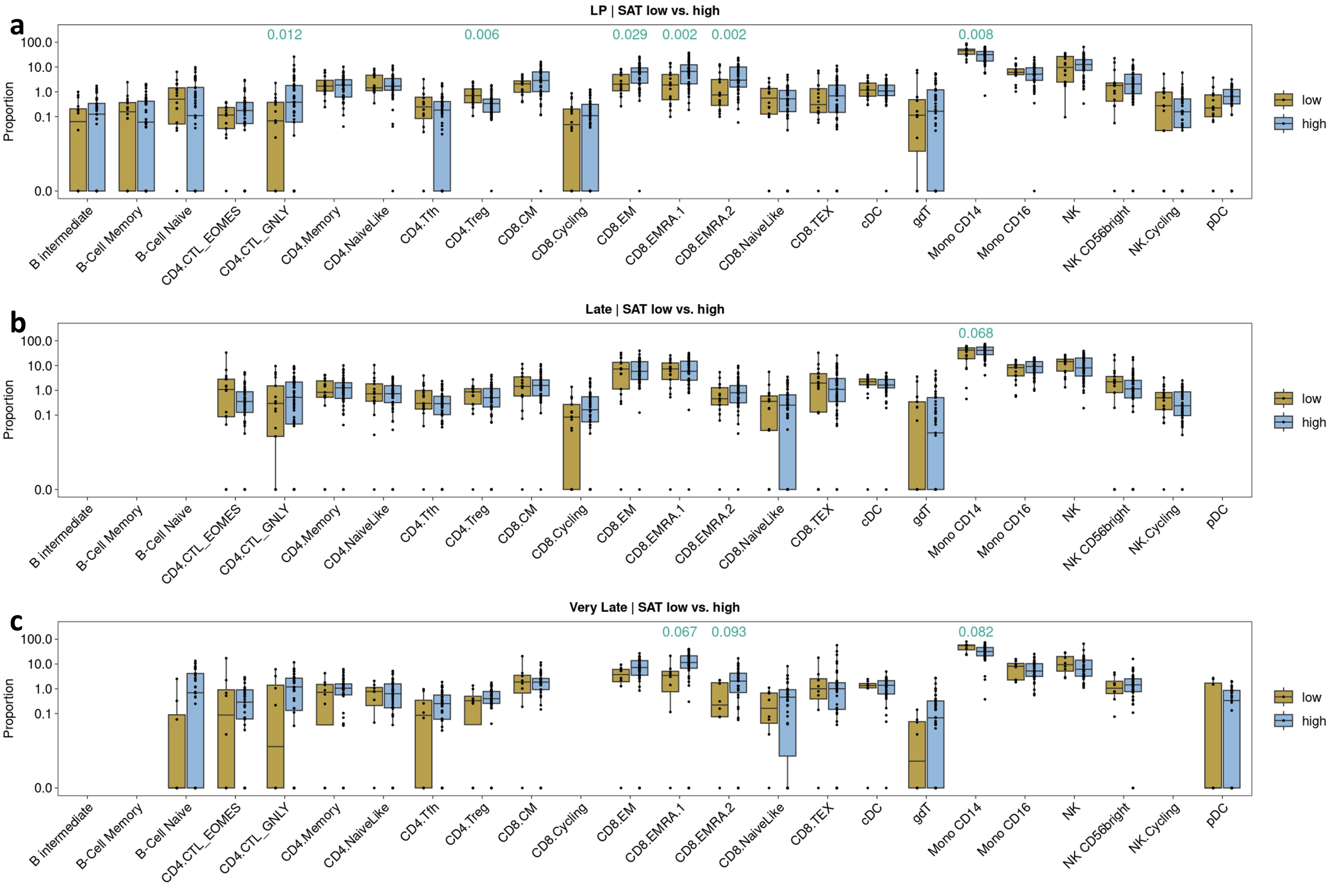
a
LP | SAT low vs. high
0.012
0.006
0.029
0.002
0.002
0.008
b
Late | SAT low vs. high
0.068
c
Very Late | SAT low vs. high
0.067
0.093
0.082
Proportion
100.0
10.0
1.0
0.1
0.0
B intermediate
B-Cell Memory
B-Cell Naive
CD4.CTL_EOMES
CD4.CTL_GNLY
CD4.Memory
CD4.NaiveLike
CD4.Tfh
CD4.Treg
CD8.CM
CD8.Cycling
CD8.EM
CD8.EMRA.1
CD8.EMRA.2
CD8.NaiveLike
CD8.TEX
cDC
gdT
Mono CD14
Mono CD16
NK
NK CD56bright
NK.Cycling
pDC
low
high

Fig S3

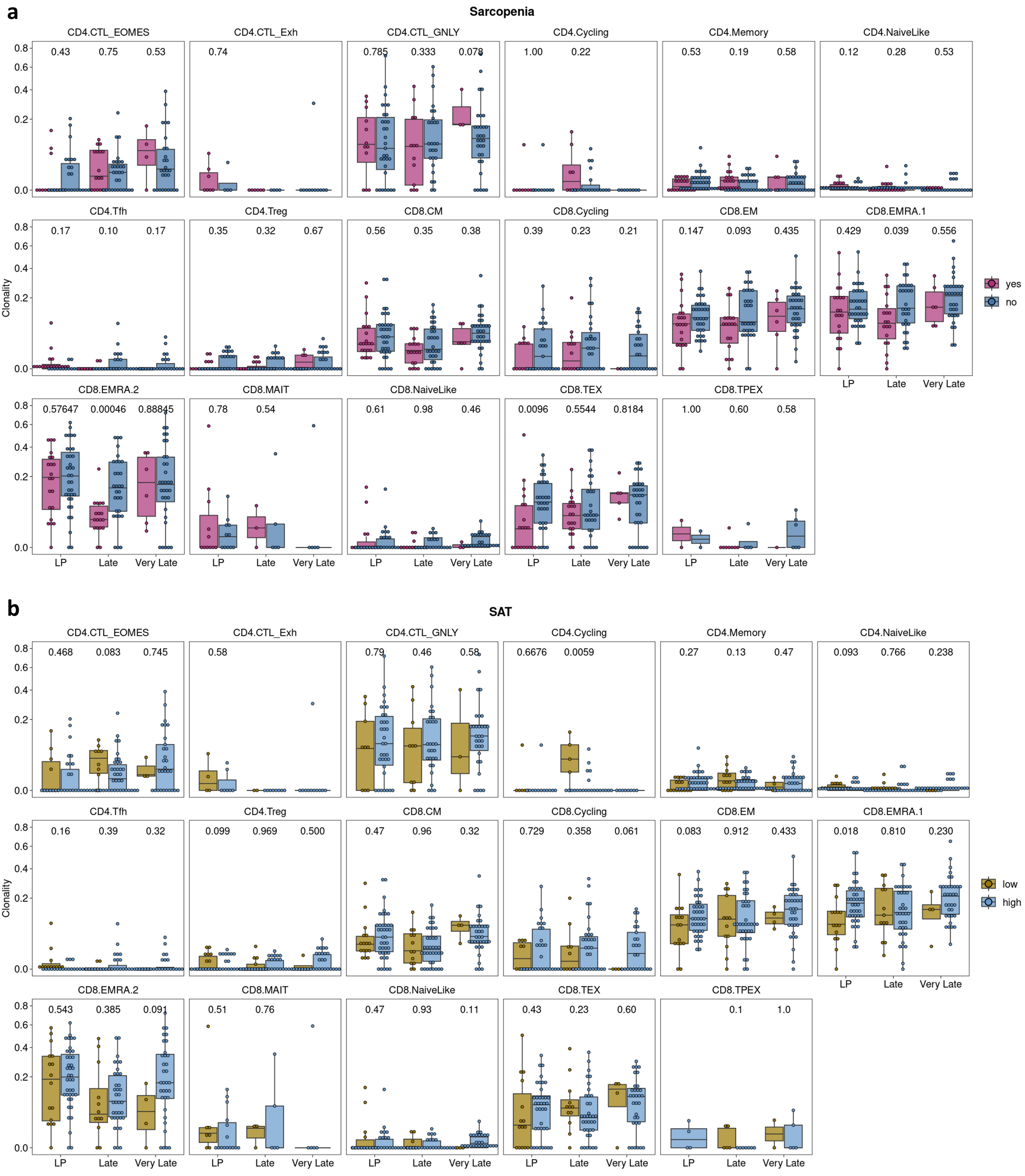
a
Sarcopenia
CD4.CTL_EOMES
0.43 0.75 0.53
CD4.CTL_Exh
0.74
CD4.CTL_GNLY
0.785 0.333 0.078
CD4.Cycling
1.00 0.22
CD4.Memory
0.53 0.19 0.58
CD4.NaiveLike
0.12 0.28 0.53
CD4.Tfh
0.17 0.10 0.17
CD4.Treg
0.35 0.32 0.67
CD8.CM
0.56 0.35 0.38
CD8.Cycling
0.39 0.23 0.21
CD8.EM
0.147 0.093 0.435
CD8.EMRA.1
0.429 0.039 0.556
CD8.EMRA.2
0.57647 0.00046 0.88845
CD8.MAIT
0.78 0.54
CD8.NaiveLike
0.61 0.98 0.46
CD8.TEX
0.0096 0.5544 0.8184
CD8.TPEX
1.00 0.60 0.58
Clonality
0.0
0.2
0.4
0.6
0.8
LP
Late
Very Late
yes
no
b
SAT
CD4.CTL_EOMES
0.468 0.083 0.745
CD4.CTL_Exh
0.58
CD4.CTL_GNLY
0.79 0.46 0.58
CD4.Cycling
0.6676 0.0059
CD4.Memory
0.27 0.13 0.47
CD4.NaiveLike
0.093 0.766 0.238
CD4.Tfh
0.16 0.39 0.32
CD4.Treg
0.099 0.969 0.500
CD8.CM
0.47 0.96 0.32
CD8.Cycling
0.729 0.358 0.061
CD8.EM
0.083 0.912 0.433
CD8.EMRA.1
0.018 0.810 0.230
CD8.EMRA.2
0.543 0.385 0.091
CD8.MAIT
0.51 0.76
CD8.NaiveLike
0.47 0.93 0.11
CD8.TEX
0.43 0.23 0.60
CD8.TPEX
0.1 1.0
Clonality
low
high

Fig. S4

a

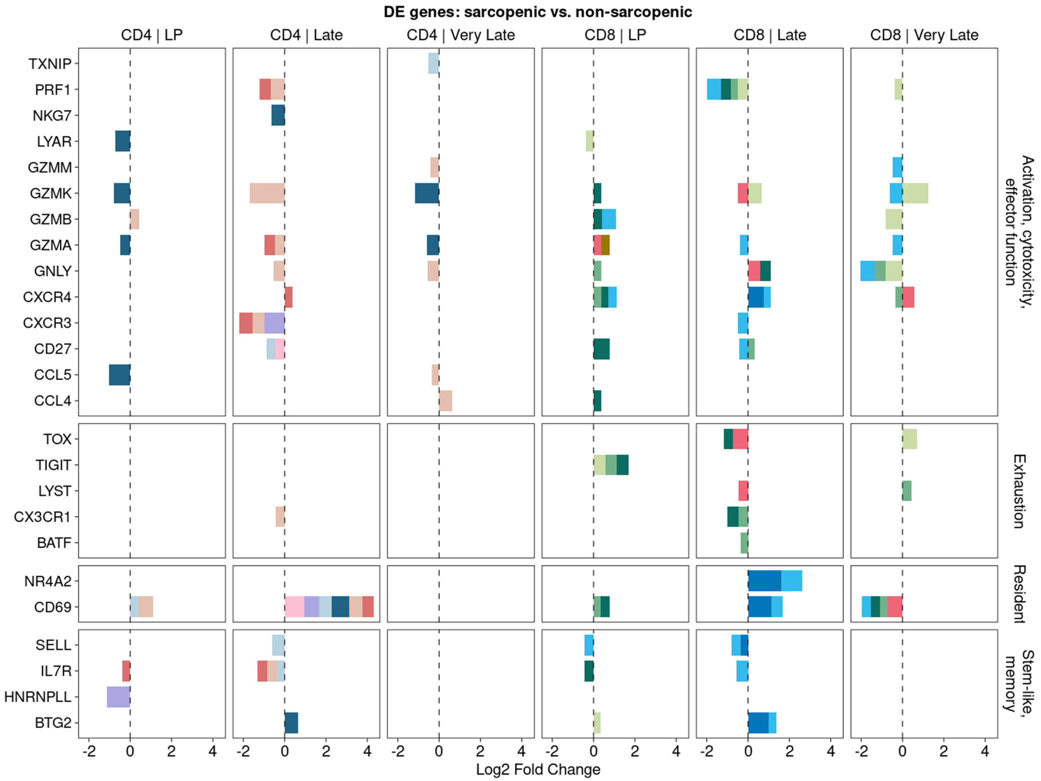
DE genes: sarcopenic vs. non-sarcopenic
CD4 | LP
CD4 | Late
CD4 | Very Late
CD8 | LP
CD8 | Late
CD8 | Very Late
TXNIP
PRF1
NKG7
LYAR
GZMM
GZMK
GZMB
GZMA
GNLY
CXCR4
CXCR3
CD27
CCL5
CCL4
TOX
TIGIT
LYST
CX3CR1
BATF
NR4A2
CD69
SELL
IL7R
HNRNPLL
BTG2
Activation, cytotoxicity, effector function
Exhaustion
Resident
Stem-like, memory
-2
0
2
4
Log2 Fold Change

Fig S5

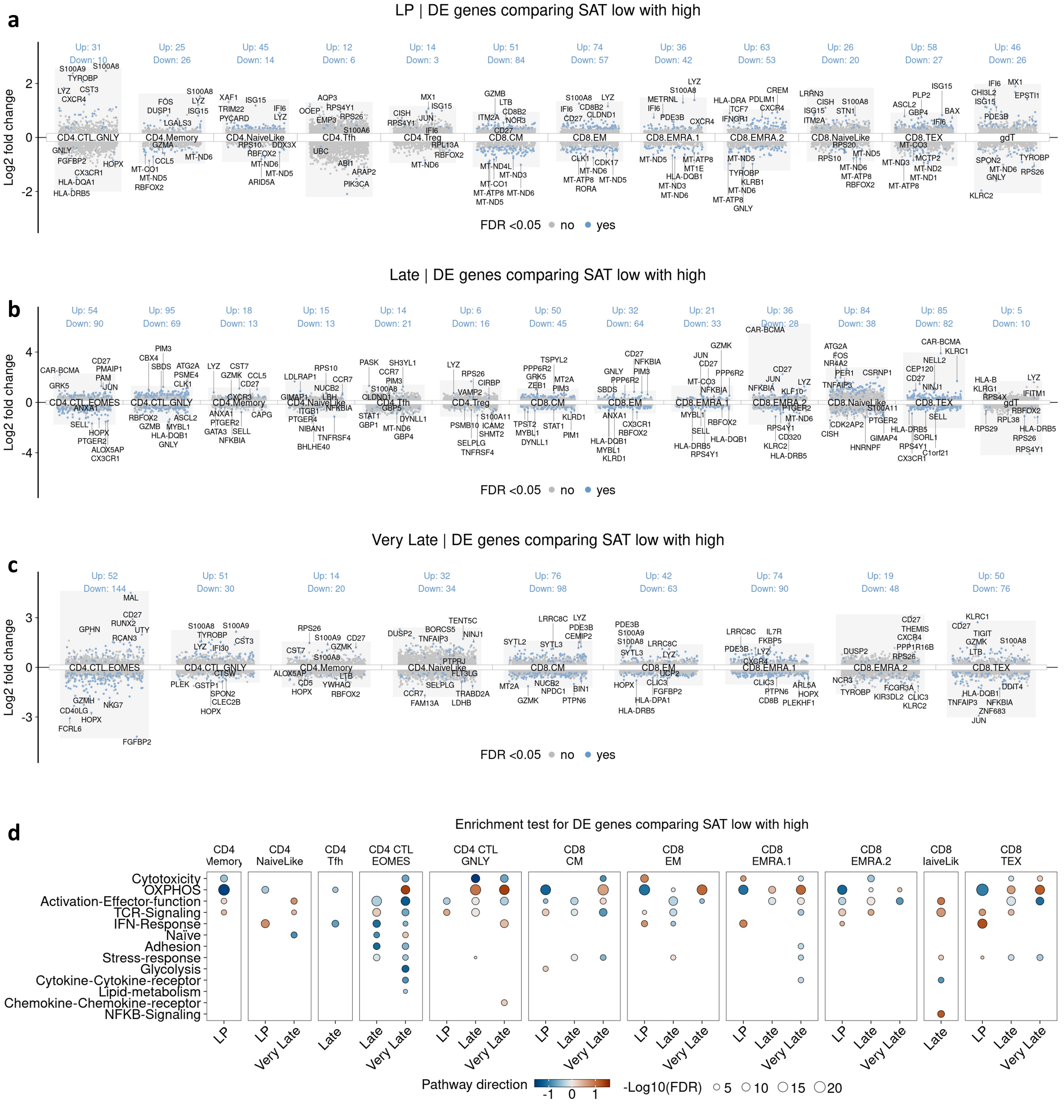
a
LP | DE genes comparing SAT low with high
Log2 fold change
Up: 31 Down: 10 CD4.CTL.GNLY
Up: 25 Down: 26 CD4.Memory
Up: 45 Down: 14 CD4.NaiveLike
Up: 12 Down: 6 CD4.Tfh
Up: 14 Down: 3 CD4.Treg
Up: 51 Down: 84 CD8.CM
Up: 74 Down: 57 CD8.EM
Up: 36 Down: 42 CD8.EMRA.1
Up: 63 Down: 53 CD8.EMRA.2
Up: 26 Down: 20 CD8.NaiveLike
Up: 58 Down: 27 CD8.TEX
Up: 46 Down: 26 gdT
FDR <0.05 no yes
b
Late | DE genes comparing SAT low with high
Log2 fold change
Up: 54 Down: 90 CD4.CTL.EOMES
Up: 95 Down: 69 CD4.CTL.GNLY
Up: 18 Down: 13 CD4.Memory
Up: 15 Down: 13 CD4.NaiveLike
Up: 14 Down: 21 CD4.Tfh
Up: 6 Down: 16 CD4.Treg
Up: 50 Down: 45 CD8.CM
Up: 32 Down: 64 CD8.EM
Up: 21 Down: 33 CD8.EMRA.1
Up: 36 Down: 28 CD8.EMRA.2
Up: 84 Down: 38 CD8.NaiveLike
Up: 85 Down: 82 CD8.TEX
Up: 5 Down: 10 gdT
FDR <0.05 no yes
c
Very Late | DE genes comparing SAT low with high
Log2 fold change
Up: 52 Down: 144 CD4.CTL.EOMES
Up: 51 Down: 30 CD4.CTL.GNLY
Up: 14 Down: 20 CD4.Memory
Up: 32 Down: 34 CD4.NaiveLike
Up: 76 Down: 98 CD8.CM
Up: 42 Down: 63 CD8.EM
Up: 74 Down: 90 CD8.EMRA.1
Up: 19 Down: 48 CD8.EMRA.2
Up: 50 Down: 76 CD8.TEX
FDR <0.05 no yes
d
Enrichment test for DE genes comparing SAT low with high
CD4 Memory
CD4 NaiveLike
CD4 Tfh
CD4 CTL EOMES
CD4 CTL GNLY
CD8 CM
CD8 EM
CD8 EMRA.1
CD8 EMRA.2
CD8 NaiveLik
CD8 TEX
Cytotoxicity
OXPHOS
Activation-Effector-function
TCR-Signaling
IFN-Response
Naïve
Adhesion
Stress-response
Glycolysis
Cytokine-Cytokine-receptor
Lipid-metabolism
Chemokine-Chemokine-receptor
NFKB-Signaling
LP
Late
Very Late
Pathway direction -1 0 1
-Log10(FDR) 5 10 15 20
