# Supplementary material for: Body composition predicts poor outcomes and reveals immunometabolic dysfunction via single‐cell profiling in anti‐BCMA CAR T‐treated myeloma
Source: Hemasphere. 2026 Mar 24;10(3):e70314. doi: 10.1002/hem3.70314 (PMC13045474; doi:10.1002/hem3.70314)
Supplement: Supplementary file 2 — supmat. [file HEM3-10-e70314-s002.docx]

Fig. [S1](#MEP_L_dmmc1) | Comparison of cell types for sarcopenic and non-sarcopenic individuals for each time point.

a-c, Differences between cell type proportions were evaluated using the speckle R package. Significant differences (unadjusted p-values) were estimated using empirical Bayes moderated t-statistics (two sided) implemented in the speckle package (*p ▒=▒ 0.1, **p ▒=▒ 0.05, ****p ▒=▒ 0.001, *****p ▒<▒ 0.0001).

Fig. [S2](#MEP_L_dmmc1) | Comparison of cell types for SAT low and SAT high groups for each time point.

a-c, Differences between cell type proportions were evaluated using the speckle R package. Significant differences (unadjusted p-values) were estimated using empirical Bayes moderated t-statistics (two sided) implemented in the speckle package (*p ▒=▒ 0.1, **p ▒=▒ 0.05, ****p ▒=▒ 0.001, *****p ▒<▒ 0.0001).

Fig. [S3](#MEP_L_dmmc1) | Differences in T-cell subtype clonality.

a, Comparison between individuals with and without sarcopenia are depicted. Difference in between SAT high and low groups are shown in b, Two-sided Wilcoxon rank sum tests were performed to calculate p-values between the groups at each time point, respectively

Fig. [S4](#MEP_L_dmmc1)▒|▒DGEA comparing cells from sarcopenic with non-sarcopenic individuals.

a, Bar plots show custom gene modules with DE genes (adjusted p-value▒<▒0.05) that functionally characterize T-cells.

Fig. [S5](#MEP_L_dmmc1) | Comparison between patients with SAT low and SAT high.

a-c, Differential gene expression analysis for T-cell subtypes and time points comparing SAT low with SAT high patients. Shown are the highest ranked (sorted by log2FC) protein-coding DE genes. A positive log2 fold change indicates upregulation in SAT low patients. d, Enrichment analysis for DE genes from all time points for T-cell subtypes. The dot plot depicts enriched T cell signatures (adjusted p-value ▒<▒ 0.05). The color indicates the pathway direction, which is the number of DE genes with a log fold change of >0 minus the number of DE genes with a log fold change of <0 divided by the square root of the number of pathway-associated genes.
