# Supplementary figures and images for: Body composition predicts poor outcomes and reveals immunometabolic dysfunction via single‐cell profiling in anti‐BCMA CAR T‐treated myeloma
Source: Hemasphere. 2026 Mar 24;10(3):e70314. doi: 10.1002/hem3.70314 (PMC13045474; doi:10.1002/hem3.70314)

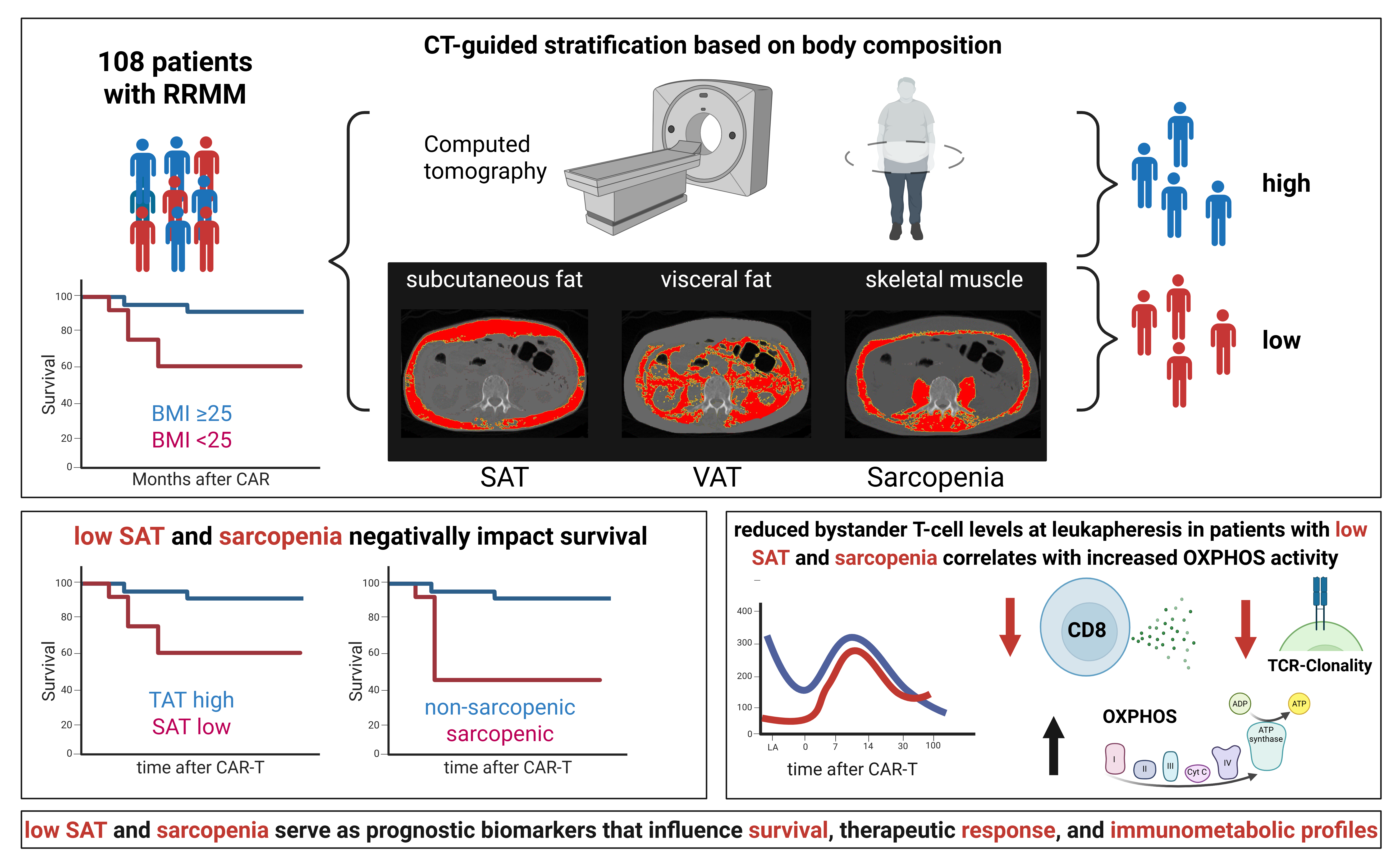

Supplement: Supplementary file 3 — Supplement. [file HEM3-10-e70314-s003.png]
